# Sympathectomy Effects on Intra-Abdominal Organ Catecholamine Levels in a Streptozotocin-Induced Diabetic Rat Model

**DOI:** 10.3390/life12122147

**Published:** 2022-12-19

**Authors:** Angélica Pérez-Juárez, Andrea Giovanna Aguirre-Pérez, Cornelio Barrientos-Alvarado

**Affiliations:** 1 Departament of Physiology, Higher School of Medicine, National Polytechnic Institute, Mexico City 11340, Mexico; 2 Faculty of Science and Mathematics, Arkansas State University, Queretaro City 75270, Mexico

**Keywords:** diabetes mellitus, sympathetic nervous system, catecholamines, streptozotocin, guanethidine

## Abstract

Diabetes mellitus (DM) is a metabolic disorder whose prevalence has continuously increased worldwide and is associated with dysfunction of the autonomic nervous system and, in particular, that of the sympathetic nervous system (SNS). The objective of this study was to analyze the interaction of DM and the SNS, building a model of sympathectomized diabetic rats to determine alterations in the content of CA (catecholamines) in different intra-abdominal organs. Sympathectomy was conducted with guanethidine (GNT). Additionally, DM was induced with STZ (Streptozotocin). Treatment with GNT decreased norepinephrine (NE) content in all analyzed tissues, with significant differences found in the paraganglia, liver, pancreas, duodenum, and heart compared to the control group. With respect to epinephrine (E), which was only found in the liver, pancreas, and heart, presenting significant differences (*p* < 0.05) in the heart, a decrease in its concentration was observed for all of the experimental groups with respect to the control. The decrease in dopamine (DA) content due to the GNT–STZ treatment was 30.1% in the heart with respect to the diabetic (STZ) group. The amount of CA in the adrenal medulla indicates the effect of sympathectomy on the GNT group where there was a significant reduction (*p* < 0.05) of DA. These findings suggest that the elimination of the sympathetic nervous system in diabetic organisms contributed to a decrease in blood glucose; likewise, an alteration in the levels of CA was observed in the different selected organs, possibly attributed to the severity, duration, and pathogenesis of the complications of acute and chronic DM.

## 1. Introduction

Diabetes mellitus (DM) is a major public health problem worldwide. Current global estimates indicate that this condition affects 463 million persons and that this number is set to escalate to 700 million by the year 2045 [1]. DM is a disorder of the metabolism of carbohydrates, proteins, and lipids associated with a relative or absolute insufficiency of the secretion of insulin and with various degrees of tissue resistance. It is typically characterized clinically by the presence of fasting hyperglycemia. In addition to the latter, the classic symptoms of DM are manifested in weight loss, polydipsia, polyphagia, and polyuria, effects reflected by the presence of hyperglycemia and insulinopenia. Some of its chronic complications include neuropathic, cardiovascular, and nephropathy problems [2].

The main factors contributing to its high prevalence and frequency include genetic predisposition, obesity, hyperinsulinemia, insulin resistance, and the abnormal cellular handling of cations [3].

Autonomic neuropathy is one of the most frequent complications in DM [4], with around 60% of persons with diabetes estimated to present some type of neuropathic alteration at the time of diagnosis, affecting the sensory, motor, and sympathetic dysfunction in 51.9% of diabetics [2,5]. The response of the sympathetic nervous system (SNS) is mediated by the release of catecholamines (CA), which act on practically all tissues, performing various functions of the organism. In the majority of cases, they participate with other hormonal and neural systems in the regulation of a multitude of different physiological processes [6].

Several studies have reported alterations of norepinephrine (NE), epinephrine (E), and dopamine (DA) levels, both in tissues and plasma, as a consequence of acute and chronic complications in DM [7,8].

DM has been reported as a pathology with important cardiovascular modifications as well [9,10]. As autonomic dysfunction occurs [11], DA modulates several physiological variables, including the increase in glucose; in addition to the latter, sympathetic hyperactivity and a greater release of cardiac NE and E have been reported [8], contributing to the occurrence of hypertension, chronic and high concentrations of NE, and influencing the pathogenesis of left ventricular hypertrophy, which is frequently accompanied by a hypertensive state [12]. Additionally, autonomic neuropathy is an important etiological factor, generating mainly digestive complications, esophageal disorders, and gastric alterations in the small intestine, colon, pancreas, and gallbladder [13]. However, there is little information on the relative susceptibility of sympathetic nerves and neurotransmitters in different peripheral tissues.

Various works have addressed the participation of the SNS and CA with the use of guanethidine (GNT) as a sympathetic denervation drug that constitutes an efficient way to study these nerve connections [14]. GNT produces a selective sympathetic blockade, inhibiting the transmission of adrenergic stimuli through peripheral nerve endings by alterations in the release and storage of norepinephrine [9,14].

The Induction of hyperglycemia in experimental models has been achieved through genetic manipulation, pancreatic surgical procedures, dietary modifications, and pharmacological induction. One of the most widely used pharmacological agents is Streptozotocin, which has a selective cytotoxicity towards pancreatic β-cells [15]. To better understand the involvement of the SNS in DM, in this study, chemical sympathectomy with GNT was performed, and subsequently, the diabetic rat model was induced with the administration of STZ, evaluating CA levels in different intra-abdominal organs. Additionally, food and water intake were analyzed and body weight (BW) was determined.

## 2. Materials and Methods

### 2.1. Animal Preparation

Male Wistar rats (*Rattus norvegicus albinus*) weighing 200–250 g were housed individually in metal cages (KT Fammai, México). All animals were maintained during a 5-day acclimatization period in an air-conditioned room, which was kept at a temperature of approximately 23 ± 2 °C and on a 12:12-h light:dark cycle (lights on from 0700 to 1900), with a relative humidity ranging from 40–70% and ventilated with filtered fresh air. The animals were allowed standard rat pellet chow (Purina de México, SA) and water ad libitum. They were randomized into four groups consisting of six animals each and were treated for eight weeks. All manipulations due to the experimental design were according to the Ethics and Biosafety Committee of the National School of Biologic Sciences, National Polytechnic Institute (zoo-14-2018). In addition, all of the procedures described in this study were set up with the Official Mexican Guidelines for Laboratory Animal Used and Care (NOM-062-ZOO-1999, México) [16].

### 2.2. Experimental Design

The animals were grouped randomly into the following four groups: (1) the control group; (2) the group with sympathectomy (GNT); (3) the diabetic group (STZ), and (4) the sympathectomized–diabetic (GNT–STZ) group, with *n* = 6 rats in each experimental group.

### 2.3. Induction of Sympathectomy

Two groups received an intraperitoneal (i.p.) dose of 50 mg/kg BW of GNT (Guanethidine monosulfate; Sigma Chemical Co., St Louis, MO, USA), five times weekly for three weeks.

### 2.4. Induction of Diabetes

After the GNT treatment, two groups of animals were induced into DM by the administration of STZ (Streptozotocin; Sigma-Aldrich, St Louis, MO, USA). This was prepared in a solution at a concentration of 9.35 mg/mL by diluting 123.75 mg of STZ in 6.6 mL of the sodium–citrate solution. Then, the solution was passed through a 0.22-μm filter and stored, protected from light, and at an average temperature of 5 °C for 3 h, in order to balance the alpha anomer. For the dilution of the STZ, 0.74 g of trisodium citrate dihydrate (Merck KGaA, Darmstadt, Germany) and 0.52 g of citric acid monohydrate (Merck KGaA, Darmstadt, Germany) were dissolved separately. The two solutions were subsequently combined in 30 mL of water and deionized at 01 M0, adjusting the pH to 4.5. DM was induced with a single injection of STZ (40 mg/kg BW, i.p.), dissolved in 0.1 M citrate buffer solution (pH 4.5), on two consecutive days. The control group was administered with citrate buffer solution under the same conditions.

DM, confirmed by the measurement of blood glucose (mg/dL) levels, was measured, without fasting, from a tail-vein blood sample. The concentration of glucose in the blood was determined with a glucometer (One Touch Basic Blood Glucose, Precision Plus MediSense) to confirm STZ injection-induced hyperglycemia.

Hyperglycemia higher than 200 mg/L was considered, in addition to the presentation of symptoms of the pathology. Measurements of BW, food consumption, and water were performed daily. All groups were sacrificed 37 days after STZ treatment.

### 2.5. Serum Biochemical Parameters

At the end of the experimental period, a sample of blood was obtained from each rat and centrifuged at 1300× *g* for 10 min. Serum was separated for the assessment of glucose, triacylglycerides, cholesterol, and albumin, which were evaluated by spectrophotometric techniques, using diagnostic kits (Spinreact de México, S.A. de C.V., Naucalpan de Juárez, Mexico) following the instructions provided by the manufacturer. The results are reported in mg/dL, except for albumin which is reported in g/dL.

### 2.6. Catecholamine (CA) Analyses

For the determination of CA, samples were taken from the paraganglia, liver, portal vein, pancreas, duodenum, heart, and adrenal medulla. Tissue samples were weighed and stored at −70 °C until further analyses. CA were analyzed by HPLC coupled to electrochemical detection [17], as briefly summarized here: tissues were homogenized in 2 mL of 0.4 N perchloric acid (PCA) and centrifuged at 3000× *g* for 10 min (Beckaman L-80); the supernatant was collected, as was CA from 500 μL, and added to columns containing 40 mg alumina, 1.5 mL 0.5 M Trizma buffer at pH 8.6, and 200 μL EDTA (5%), with 100 μL 3,4-dihydroxybenzylamine hydrobromide (DHBA, 100 ng mL^−1^) as internal standard.

Using 30-μL aliquots of the solution, the concentrations of CA were analyzed by HPLC coupled to electrochemical detection (ESA, Bedford, MA, USA), reversed-phase (column C-18, 15 × 4.6 mm). The first detector potential was set at −200 mV and the second at +200 mV. The mobile phase was composed of 1 mM heptanosulfonic acid sodium salt, 1 mM EDTA, 0.15 M NaH2PO4·H2O, and 6% methanol at pH 3.6, adjusted with phosphoric acid and at a 0.4-mL flow rate per min. CA was identified by the comparison of retention times to those of known standards and quantified by using the peak–area ratio related to DHBA, with the internal standard used to correct for recovery. The results are expressed in ng/g/tissue and, for the adrenal medulla, in μg/gland.

### 2.7. Statistical Analysis

Glucose, BW, food intake, water consumption, and concentration of CA are expressed as mean values and by the corresponding standard errors ± the standard error of the mean (SEM). Treatment effects were analyzed by one-way ANOVA followed by the Tukey HSD post-hoc test for mean comparisons, employing SPSS Statistical Software version 22.0 for Windows (Chicago, IL, USA). The level of significance was considered at *p* < 0.05.

## 3. Results

### 3.1. Effect of Chemical Sympathectomy and DM on Blood Glucose, Triacylglycerides, Cholesterol, and Albumin

The effect of GNT and DM was observed in blood glucose concentrations. The values are expressed as the mean ± SEM in each experimental group and are illustrated in Table 1.

For animals treated with GNT, they did not present significant differences with respect to the control group, with blood glucose concentrations of around 100 mg/dl observed in both experimental groups. However, the two groups that received STZ developed severe hyperglycemia, presenting values above 400 mg/dL of blood glucose which were statistically different (*p* < 0.001) with respect to the control group and the sympathectomized group (GNT). Sympathectomy in the diabetic group (GNT–STZ) reflected a decrease in glycemia (20.6%) compared to the diabetic group (STZ).

The induction of DM (STZ and GNT-STZ groups) was associated with a significant (*p* < 0.05) increase in cholesterol levels compared to the control (63.33 ± 0.9 mg/dL) and GNT (71.15 ± 5.9 mg/dL) group. As can be observed in Table 1, only in the STZ group was there a significant (*p* < 0.05) increase in triacylglycerol concentration (198.42 ± 3.2 mg/dL) compared to all experimental groups, with no significant differences found in the remaining three groups compared to the control.

Serum albumin concentrations for the groups STZ and GNT-STZ, presented significant decreases (*p* < 0.05) with respect to the values of the control group (3.70 ± 0.15 g/dL) and GNT group (3.59 ± 0.19 g/dL) (Table 1).

### 3.2. Effect of Chemical Sympathectomy and DM on Body Weight and Food and Water Intake

**Figure 1:**
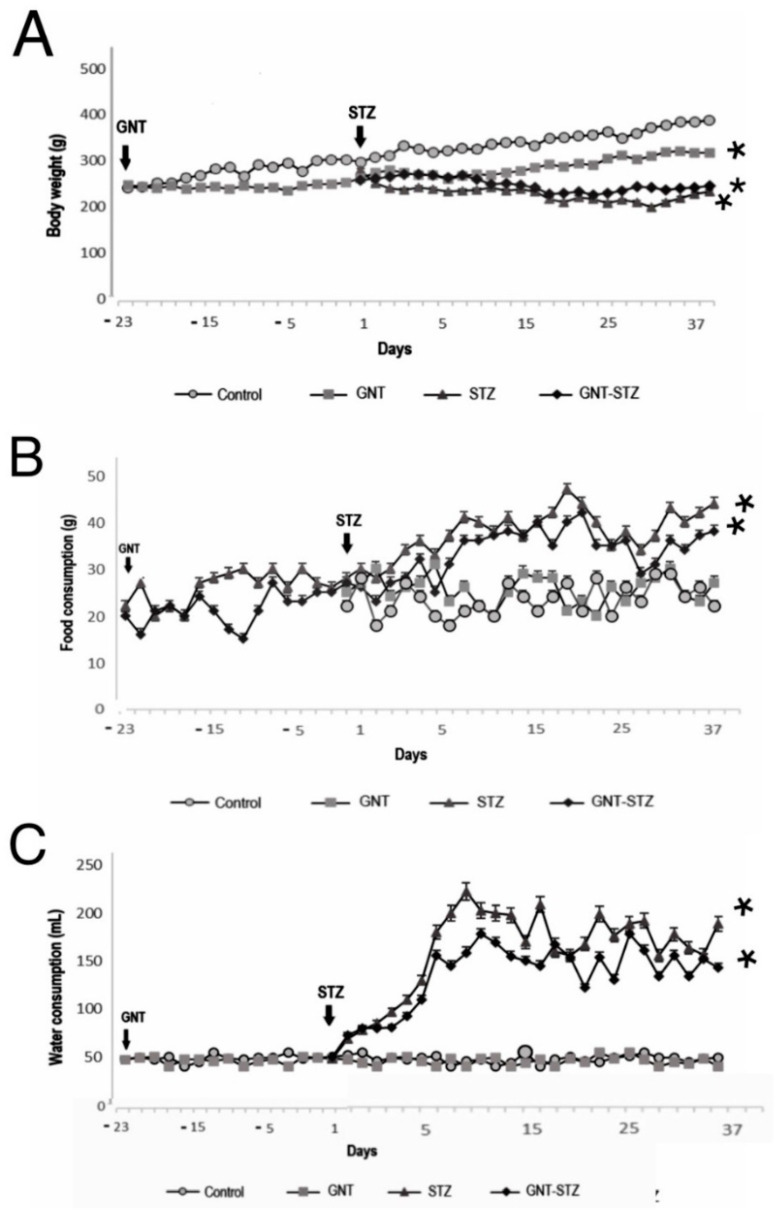
Body weight (**A**), food consumption (**B**), and water intake (**C**) in the control group and the groups treated with GNT, STZ, and a combination of GNT−STZ (*n* = 6). The represented values are the mean (± SEM), * *p* < 0.05 vs. the control group.

Regarding weight gain, it was observed that at the end of the experiment in the sympathectomized group (GNT), the animals had a BW loss of 11.47% (41.23 ± 1.9 g) compared to the control group. The animals treated with the diabetogenic agent (STZ) exhibited a marked decrease in BW; it could be observed that the weight of the control rats (388.1 ± 2.3 g) was greater than that of the DM-induced rats, with the STZ group having a BW of 239.4 ± 1.4 g. Finally, the GNT–STZ group presented a BW of 246.3 ± 2.2 g, a statistically significant difference for both diabetic groups (*p* < 0.05) with respect to the control group (Figure 1A). Secondly, with respect to food intake, no significant differences were found between the control group and the sympathectomized group. However, the groups treated with STZ (Figure 1B) presented a significant difference (*p* < 0.05) with respect to these two groups. No significant differences were observed in water intake between the sympathectomized group (GNT) and the control animals, with a daily consumption of approximately 50 mL for both groups. However, a 400% increase in water intake was found for the STZ and GNT–STZ groups after DM induction, being statistically significant with respect to the control group and the GNT group (Figure 1C).

### 3.3. Concentration of CA in the Organs of the Splanchnic Area of Control, Guanetinized and Diabetic Rats

**Figure 2:**
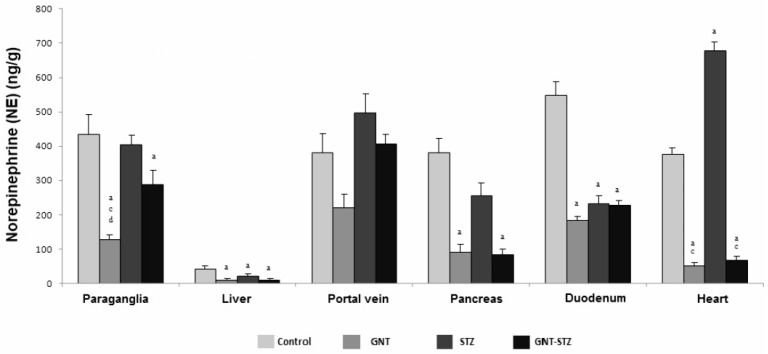
NE concentrations (ng/g) in different intra-abdominal tissues, including the paraganglia, liver, portal vein, pancreas, duodenum, and heart of control animals, the sympathectomized group (GNT), the group administered with STZ, and the group administered with the combination of GNT–STZ. The data are the mean ± SEM (*n* = 6), ^a^ *p* < 0.05 compared with the control group, ^c^ *p* < 0.05 compared with the STZ group, and ^d^ *p* < 0.05 compared with the GNT–STZ group.

Treatment with GNT decreased NE content in all analyzed tissues, with significant differences found in the paraganglia, liver, pancreas, duodenum, and heart (*p* < 0.05) compared to the control group. However, with sympathectomy in the portal vein, no significant difference was observed, although the concentration of NE decreased by 41.8% compared to that of the control animals. The levels of NE in the heart for the STZ group (677.8 ± 4.6 ng/g) were statistically significant (*p* < 0.05) with respect to all of the experimental groups, highlighting an increase of 55% with regard to the control group. For the group treated with GNT and induced to DM (GNT–STZ), there were significant differences (*p* < 0.05) in terms of a decrease in the concentration of NE in the paraganglia, liver, pancreas, and heart compared to control rats (Figure 2).

**Figure 3:**
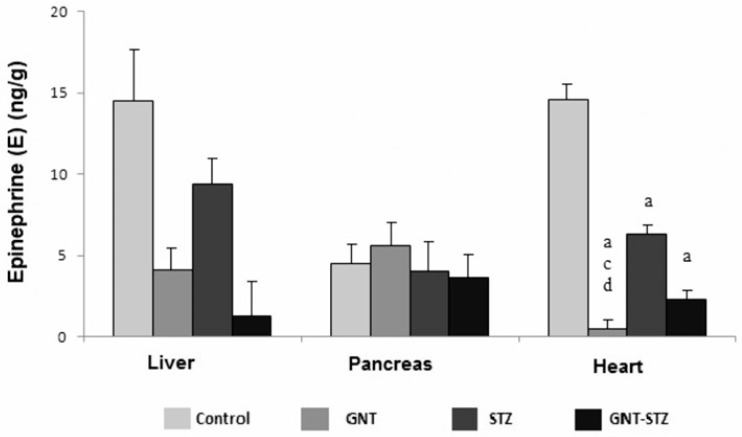
Effect of the administration of GNT and STZ on E concentrations (ng/g) in the liver, pancreas, and heart. Values are expressed as the mean ± SEM in each experimental group (*n* = 6), ^a^ *p* < 0.05 compared with the control group, ^c^ *p* < 0.05 compared with the STZ group, and ^d^ *p* < 0.05 compared with the GNT–STZ group.

Epinepheline (E) was the least abundant CA, observed only in the liver, pancreas, and heart, and presenting the lowest concentrations with respect to NE and DA. Sympathectomy and induction of DM decreased E concentrations in the liver with respect to control animals. In the case of the pancreas, no significant differences were observed; however, for the heart, all of the groups treated with GNT and STZ were statistically different (*p* < 0.05) from the control group (Figure 3).

**Figure 4:**
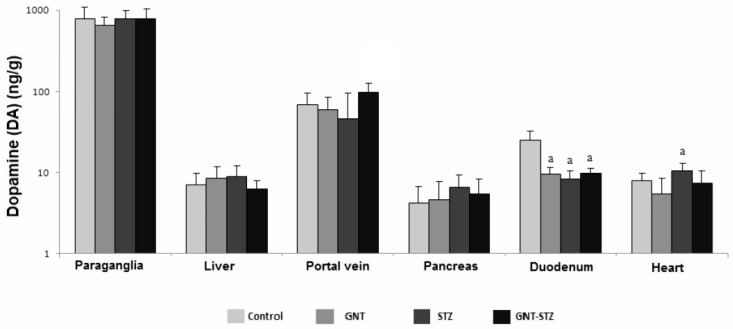
DA concentrations (ng/g) in different tissues of male Wistar rats, including the paraganglia, liver, pancreas, duodenum, and heart of control animals, the sympathectomized group (GNT), the group administered with STZ, and the group administered with the combination of GNT–STZ. The data are the mean ± SEM, (*n* = 6), ^a^ *p* < 0.05 compared with the control group.

There were no significant variations in DA in the paraganglia, liver, and pancreas (Figure 4). The different sympathectomized and diabetic animals’ (GNT, STZ, and GNT–STZ) DA content in the duodenum significantly decreased (*p* < 0.05) compared with control animals. DA levels (10.6 ± 1.1 ng/g) in the heart for diabetic animals (STZ) increased significantly (*p* < 0.05) by 27.7% compared to control values (8.3 ± 1.1 ng/g).

### 3.4. Concentration of CA in Adrenal Medulla

**Figure 5:**
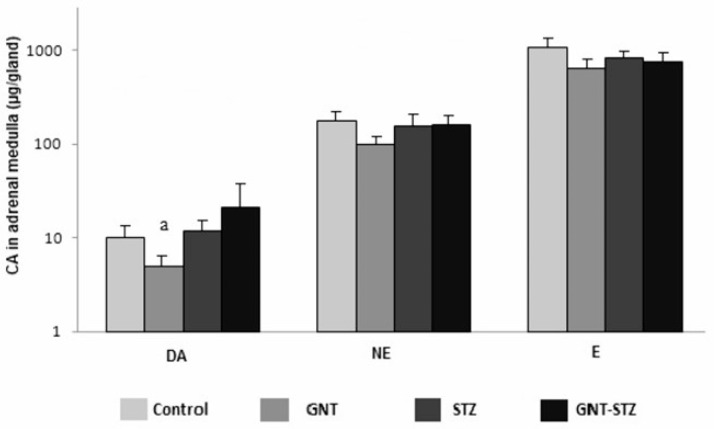
DA, NE, and E concentrations (μg/gland) in the adrenal medulla of the control group, sympathectomized (GNT) group, diabetic (STZ) group, and the interaction of sympathectomy and diabetes (GNT–STZ) group, male rats (*n* = 6 for each group). Values are the mean ± SEM. ^a^ *p* < 0.05 compared with the control group.

In the present study, we determined the amount of CA in the adrenal medulla. The results indicate the effect of sympathectomy on the GNT group where there was a significant reduction (*p* < 0.05) in the amount of DA (5.3 ± 1.2) compared to the control group. The amount of NE in the groups treated with GNT and STZ presented similar values to those of the control; thus, no significant differences were found. Similarly, the same behavior was observed for the values of E, with no significant differences found among the different experimental groups (Figure 5).

## 4. Discussion

STZ-induced experimental diabetes has been documented to manifest characteristics similar to those of DM in humans [18], reflecting metabolic alterations due to the relative or absolute absence of insulin, which exhibits a degradation of β-cells [15].

In the present study, we observed clinical manifestations, including hyperglycemia, polyphagia, polyuria, and polydipsia accompanied by weight loss, which was observed in adult rats in the STZ treatment, indicating the irreversible destruction of Langerhans islet cells [19].

In this regard, DM, a disorder of multiple etiologies characterized by chronic hyperglycemia and high levels of glycemia (>400 mg/dL) was observed in the diabetic groups, this being significant compared to the other groups. This is in agreement with what was reported by some researchers, who noted that, immediately after administering STZ, the animals presented hyperglycemia [20]. On the other hand, the elimination of SNS with GNT in the diabetic group (GNT–STZ) contributed to a slight decrease in blood glucose compared to the group treated only with STZ. This is consistent with what has been previously reported, that blood glucose levels may be decreased by denervation of the SNS in target organs, such as the liver, pancreas, skeletal muscle, and adipose tissue, caused by the inhibition of acute catabolic actions (i.e., glycogenolysis and lipolysis), while DM begins with impaired carbohydrate metabolism which then commits to lipids and proteins. It is known that cholesterol and triglycerides are increased in diabetic animals, which is consistent with the present study [21,22]. Albumin, the most abundant protein in serum, possesses antioxidant properties, and a decrease in this protein can result in attenuation in the regulation of oxidative stress in cells [23]. These were reflected in the STZ and GNT-STZ groups, where the level of albumin was lowest compared to the control and GNT groups.

The SNS is critical in the control of daily energy expenditure through the regulation of resting metabolic rate and thermogenesis in response to different physiological stimuli, energy states, food intake, carbohydrate consumption, and hyperinsulinemia [24,25].

However, BW in the sympathectomized (GNT) group was not affected (Figure 1A). Thus, it could be speculated that the sensitization of adrenergic receptors to sympathectomy could be sufficient for the partial recovery of sympathetic functionality in the control of eating behavior, therefore regulating BW [26,27,28].

One of the main parameters affected in diabetic patients is BW; it has been described that there is a reduced ability to gain body mass in DM [28].

The foregoing is consistent with the present study, where the effect of DM on this parameter in the diabetic groups (STZ and GNT–STZ) could be observed to present progressive BW loss throughout the experiment, a factor caused by the inefficient supply of cellular glucose, resulting in increased catabolism and low anabolism [29,30] (Figure 1A). This weight loss is also associated with the excessive diuresis characteristic of diabetic conditions [21,31]. In addition, the presence of diarrhea was observed in diabetic animals, coinciding with what was reported as one of the most common disorders in diabetic patients, attributed as a consequence of neuronal alteration and loss of adrenergic tone. In addition, there are also alterations in gastrointestinal motility and the control of electrolytes and water absorption in the intestinal epithelium [21].

On the other hand, the resulting hyperglycemia in the diabetic groups causes glycosuria and dehydration due to osmotic diuresis, giving rise to the presence of polydipsia [31] (Figure 1B). To deal with intracellular glucose deficiency, the appetite is stimulated (Figure 1C), performing gluconeogenesis, and the energy supply is thus maintained by the metabolism of proteins and fats [25], resulting in weight loss, and confirmed by the eating behavior in the STZ and GNT–STZ groups.

The SNS through CA plays a vital role in the regulatory mechanisms of blood pressure, sodium balance, and the maintenance of the homeostatic state.

Sympathectomy is considered an efficient model that depresses sympathetic postganglionic responses [9,29,32], this being consistent with the results obtained, where an evident decrease in NE concentration was observed in all of the tissues analyzed. The reduction in NE may be determined in that it is found mainly peripherally, lodged in the nerve terminals; therefore, the content of NE in a particular tissue will reflect its degree of sympathetic innervation. In the case of paraganglia, the concentration of NE was affected by the administration of GNT. Despite the fact that its secretory characteristics are similar to those of the adrenal medulla [33,34], a decrease in its values was found in the sympathectomized groups (GNT and GNT–STZ).

With respect to the liver, the latter presented the lowest concentrations of NE for the groups treated with GNT and STZ, this being significant with respect to the control group, with these low levels attributed to its degree of sympathetic innervation, as well as to its efficient role in removing concentrations of NE [32,35]. Sympathetic hyperactivity could be observed in the group treated with STZ, giving rise to a significant increase in NE in the portal vein and heart, highlighting what has been reported in different studies on CA in terms of its increase being clinically relevant, damaging different target organs related to obesity, including cardiac function and structure, endothelial dysfunction, hypertension, kidney damage, and oxidative stress, among others [31,36,37]. This is consistent with what has been reported, presenting, in the diabetic group (STZ), a significant increase of 57.5% of NE in the heart with respect to the control group, a factor associated with increased sympathetic activity which increases the release of systemic NE. Therefore, the overactivation of the SNS in DM exerts unfavorable effects, such as cardiac hypertrophy, arterial remodeling, and endothelial dysfunction in the cardiovascular system [38,39].

The content of E in peripheral tissues is not constant and occurs in small amounts corresponding to 2–15% of the total amount of catecholamine [40]. The latter was consistent with the present work, in which we only determined E in the liver, pancreas, and heart, finding significant differences (*p* < 0.05) only in the heart, where all groups treated with GNT and STZ presented lower amounts than the control group (Figure 3).

In the case of DA, the concentrations were not consistent in the different groups studied; these discrepancies are explained by the selected tissues, coupled with the low or minimal levels of this catecholamine with respect to NE, perhaps due to the rapid transformation of DA into NE [33,41], which would cause the cells that contained DA as a precursor to not accumulate large amounts of it [42], coinciding with what was reported for the rat heart, which has low levels of DA [43].

Paraganglia are noteworthy in this respect because this is the only tissue in which the DA concentration increased in the diabetic group as well as in the two sympathectomized groups, although it was not statistically significant. It should be mentioned that this behavior possibly takes place as a compensatory increase, considering this tissue as an extra-adrenal source of CA, in that the few reports on increases in CA in different organs in the face of chronic treatment with GNT are controversial [34]. It is worth mentioning that, in the liver and pancreas of the different experimental groups, DA did not demonstrate a significant alteration, which suggests that, after the administration of GNT, the baseline amount of this amine is so low that it can be sustained by the permanence of the few sympathetic fiber cells after sympathectomy, or that DA is being synthesized by fibers resistant to treatment (dopaminergic nerve fibers or non-nerve cells) [41,43].

The CA concentration for the adrenal medulla can be observed in Figure 5. The DA participates as a precursor for the synthesis of NE, and this, in turn, is a precursor of E [34,42,44]. This is consistent with the present study where the DA presented lower concentrations with respect to the other CA. However, there was a significant decrease in the sympathectomized group (GNT) with respect to the control. The hyperactivity of the SNS is supported by an increase in the levels of NA and E in the plasma and specific organs [25], which agrees with the results obtained in the levels of NE and E present in the adrenal medulla; despite this, in regard to NE, no significant differences were observed between the groups studied. Although E presented the highest levels of CA analyzed, this can be attributed to the fact that E is the main CA secreted by the adrenal medulla [40]. Acute or chronic catecholaminergic excitation can trigger different complications, mainly the high concentrations of NE and E in DM, which are the result of metabolic, vascular, cardiac, and renal conditions, as well as other clinical manifestations [20,29,32].

## 5. Conclusions

In conclusion, the present study suggests that the elimination of the SNS in diabetic organisms decreases blood glucose, cholesterol, and albumin. In addition, it could be speculated that the sensitization of adrenergic receptors to sympathectomy could be sufficient for the partial recovery of sympathetic functionality in the control of eating behavior, therefore regulating BW. Likewise, an alteration in the levels of CA in the different selected organs and a decrease in the levels of NA and ADR in the sympathectomized diabetic group (GNT-STZ) was observed with respect to the diabetic group (STZ), which could possibly be attributed to the severity, duration, and pathogenesis of the complications of acute and chronic DM.

## Figures and Tables

**Table 1:** Serum metabolites in the control group and the groups treated with GNT, STZ, and a combination of GNT–STZ.

Group	Glucose (mg/dL)	Triacylglycerol (mg/dL)	Cholesterol (mg/dL)	Albumin (g/dL)
Control	98 ± 0.9 ^b^	129.74 ± 2.8 ^b^	63.33 ± 0.9 ^b^	3.70 ± 0.15 ^a^
GNT	96 ± 1.2 ^b^	139.66 ± 7.4 ^b^	71.15 ± 5.9 ^b^	3.59 ± 0.19 ^a^
STZ	508 ± 2.8 ^a^	198.42 ± 3.2 ^a^	172.73 ± 2.4 ^a^	3.04 ± 0.28 ^b^
GNT-STZ	411 ± 1.9 ^a^	172.16 ± 9.6 ^a,b^	164.32 ± 7.0 ^a^	3.11 ± 0.13 ^b^

Parameters are expressed as the mean ± standard error of the mean (SEM) (*n* = 6). Different letters indicate significant differences among the means (*p* < 0.05).

## Data Availability

Not applicable.

## References

[1] Saeedi P., Petersohn I., Salpea P., Malanda B., Karuranga S., Unwin N., Colagiuri S., Guariguata L., Motala A.A., Ogurtsova K. (2019). IDF Diabetes Atlas Committee. Global and regional diabetes prevalence estimates for 2019 and projections for 2030 and 2045: Results from the International Diabetes Federation Diabetes Atlas, 9th edition. Diabetes Res. Clin. Pract..

[2] Zhang X., Yang X., Sun B., Zhu C. (2021). Perspectives of glycemic variability in diabetic neuropathy: A comprehensive review. Commun. Biol..

[3] Nalysnyk L., Hernandez-Medina M., Krishnarajah G. (2010). Glycaemic variability and complications in patients with diabetes mellitus: Evidence from a systematic review of the literature. Diabetes Obes. Metab..

[4] Verrotti A., Prezioso G., Scattoni R., ChiarellI F. (2014). Autonomic neuropathy in diabetes mellitus. Front. Endocrinol..

[5] Jyotsna V.P., Sahoo A., Sreenivas V., Deepak K.K. (2009). Prevalence and pattern of cardiac autonomic dysfunction in newly detected type 2 diabetes mellitus. Diabetes Res. Clin. Pract..

[6] Paravati S., Warrington S.J. (2019). Physiology, Catecholamines.

[7] Guy D.A., Sandoval D., Richardson M.A., Tate D., Flakoll P.J., Davis S.N. (2005). Differing physiological effects of epinephrine in type 1 diabetes and nondiabetic humans. Am. J. Physiol. Endocrinol. Metab..

[8] Thackeray J.T., Beanlands R.S., Dasilva J.N. (2012). Altered sympathetic nervous system signaling in the diabetic heart: Emerging targets for molecular imaging. Am. J. Nucl. Med. Mol. Imaging.

[9] Dhalla N.S., Ganguly P.K., Bhullar S.K., Tappia P.S. (2019). Role of catecholamines in the pathogenesis of diabetic cardiomyopathy 1. Can. J. Physiol. Pharmacol..

[10] Motataianu A., Barcutean L., Maier S., Balasa A., Stoian A. (2020). Cardiac Autonomic Neuropathy in Diabetes Mellitus Patients Are We Aware of the Consequences?. Acta Marisiensis Ser. Med..

[11] Vinik A.I., Maser R.E., MitchelL B.D., Freeman R. (2003). Diabetic autonomic neuropathy (Technical Review). Diabetes Care.

[12] Grassi G., Mark A., Esler M. (2015). The sympathetic nervous system alterations in human hypertension. Circ. Res..

[13] Bagyánszki M., Bódi N. (2012). Diabetes-related alterations in the enteric nervous system and its microenvironment. World J. Diabetes.

[14] Blommaart P.J., Ferwerda G., Kodde A., Tytgat G.N., Boeckxstaens G.E. (1999). Nicotic acetylcholine receptor blocking effect of guanethidine in the rat gastric fundus. J. Pharmacol..

[15] Akbarzadeh A., Norouzian D., Mehrabi M.R., Jamshidi S.H., Farhangi A., Verdi A.A., Mofidian S.M., Rad B.L. (2007). Induction of diabetes by Streptozotocin in rats. Indian J. Clin. Biochem..

[16] NORMA Oficial Mexicana NOM-062-ZOO-1999 (1999). Especificaciones Técnicas para La Producción, Cuidado y Uso de Los Animales de Laboratorio. http://www.fmvz.unam.mx/fmvz/principal/archivos/062ZOO.PDF.

[17] Eriksson B.M., Persson B.A. (1982). Determination of catecholamines in rat heart tissue and plasma samples by liquid chromatography with electrochemical detection. J. Chromatogr..

[18] Veeranjaneyulu C., Subrahmanyam G.B. (2016). Rediscovered the induction of diabetogenic agents in the experimental animal model: Review. Int. J. Appl. Biol. Pharm. Technol..

[19] Takada J., Machado M.A., Peres S.B., Brito L.C., Borges-Silva C.N., Costa C.E., Fonseca-Alaniz M.H., Andreotti S., Lima F.B. (2006). Neonatal streptozotocin-induced diabetes mellitus: A model of insulin resistance associated with loss of adipose mass. Metabolism.

[20] Thackera Y.J., Radziuk J., Harper M.E., Suuronen E., Ascah K., Beanlands R., Dasilva J. (2011). Sympathetic nervous dysregulation in the absence of systolic left ventricular dysfunction in a rat model of insulin resistance with hyperglycemia. Cardiovasc. Diabetol..

[21] Chang C.J., Wu J.S., Lu F.H., Liu I.M., Chi T.C., Cheng J.T. (1988). Sympathetic hyperactivity in wistar rats with insulin-resistence. J. Auton. Nerv. Syst..

[22] Wang J., Wernette C.M., Judd R.L., Huggins K.W., White B.D. (2008). Guanethidine treatment does not block the ability of central leptin administration to decrease blood glucose concentrations in streptozotocin-induced diabetic rats. J. Endocrinol..

[23] Oettl K., Stauber R.E. (2007). Physiological and pathological changes in the redox state of human serum albumin critically influence its binding properties. Br. J. Pharmacol..

[24] Esler M., Rumantir M., Wiesner G., Kaye D., Hasting J., Lambert G. (2001). Sympathetic nervous system and insulin resistance: From obesity to diabetes. Am. J. Hypertens..

[25] Thorp A.A., Schlaich M.P. (2015). Relevance of sympathetic nervous system activation in obesity and metabolic syndrome. J. Diabetes Res..

[26] Brest A.N., Novack Kasparian-Hratch P., Moyer J.H. (1962). Guanethidine. Chest.

[27] Demas G.E., Bartness T.J. (2001). Novel method for localized, functional sympathetic nervous system denervation of peripheral tissue using guanethidine. J. Neurosci. Methods.

[28] Grassi G., Seravalle G., Colombo M., Bolla G., Cattaneo B.M., Cavagnini F., Mancia G. (1998). Body weight reduction, sympathetic nerve traffic, and arterial baroreflex in obese normotensive humans. Circulation.

[29] Lambert E., Sari C.I., Dawood T., Nguyen J., Mcgrane M., Eikelis N., Chopra R., Wong C., Chatzivlastou K., Head G. (2010). Sympathetic nervous system activity is associated with obesity-induced subclinical organ damage in young adults. Hypertension.

[30] Bellush L.L., Reid S.G., North D. (1991). The functional significance of biochemical alterations in streptozotocin-induced diabetes. Physiol. Behav..

[31] Kassab S., Kato T., Wilkins F.C., Chen R., Hall J.E., Granger J.P. (1995). Renal denervation attenuates the sodium retention and hypertension associated with obesity. Hypertension.

[32] Eisenhofer G., Aneman A., Hooper D., Holmes C., Goldstein D.S., Friberg P. (1995). Production and metabolism of dopamine and norepinephrine in mesenteric organs and live of swine. Am. J. Physiol..

[33] Eisenhofer G., Kopin I.J., Goldstein D.S. (2004). Catecholamine metabolism: A contemporary view with implications for physiology and medicine. Pharmacol. Rev..

[34] Ross L.L., Pylipiw A., Mccarthy-Cosio L. (1985). The effects of neonatal guanethidine treatment on the adrenal medulla of the rat. Annu. Meet. Soc. Neurosci..

[35] Johnson E.M., O’Brien F. (1976). Evaluation of the permanet sympathectomy produced by the administration of guanethidine to adult rats. J. Pharmacol. Exp. Ther..

[36] Hall J.E., Brands M.W., Hildebrandt D.A., Kuo J., Fitzgerald S. (2000). Role of sympathetic nervous system and neuropeptides in obesity hypertension. Braz. J. Med. Biol. Res..

[37] Tangvarasittichai S. (2015). Oxidative stress, insulin resistance, dyslipidemia and type 2 diabetes mellitus. World J. Diabetes.

[38] Frontoni S., Bracaglia D., Gigli F. (2005). Relationship between autonomic dysfunction, insulin resistance and hypertension, in diabetes. Nutr. Metab. Cardiovasc. Dis..

[39] Grassi G., Seravalle G. (2006). Autonomic imbalance and metabolic syndrome: Unravelling interactions, mechanisms and outcomes. J. Hypertens..

[40] Berecek K.H., Brody M.J. (1982). Evidence for a neurotransmitter role for epinephrine derived from the adrenal medulla. Am. J. Physiol..

[41] Fuller R.M., Snoddy H.D., Perry K.W. (1982). Dopamine accumulation alter dopamine B-hidroxilase inhibition in rat Herat as an index of norepinephrine turnover. Life Sci..

[42] Pivonello R., Ferone D., DE Herder W.W., De Krijger R.R., Waaijers M., Mooij D.M., Van-Koetsveld P.M., Barreca A., De Caro M.L., Lombardi G. (2004). Dopamine receptor expression and function in uman normal adrenal gland and adrenal tumors. J. Clin. Endocrinol. Metab..

[43] Sonne J., Goyal A., Lopez-Ojeda W. (2022). Dopamine.

[44] Andén N.E., Grabowska-Andén M., Schwieler J. (1989). Transfer of DOPA from the sympatho-adrenal system to the pancreas, liver and kidney via the blood circulation. Acta Physiol. Scand..

